# All-Solid-State Beam Steering via Integrated Optical Phased Array Technology

**DOI:** 10.3390/mi13060894

**Published:** 2022-06-03

**Authors:** Shi Zhao, Jingye Chen, Yaocheng Shi

**Affiliations:** State Key Laboratory for Modern Optical Instrumentation, Center for Optical and Electromagnetic Research, International Research Center for Advanced Photonics, Ningbo Research Institute, College of Optical Science and Engineering, Zhejiang University, Hangzhou 310058, China; zhaoshi@zju.edu.cn (S.Z.); yaocheng@zju.edu.cn (Y.S.)

**Keywords:** beam steering, optical phased array, micro-electromechanical system, liquid crystals, photonic integrated chip, silicon photonics, diffraction

## Abstract

Light detection and ranging (LiDAR), combining traditional radar technology with modern laser technology, has much potential for applications in navigation, mapping, and so on. Benefiting from the superior performance, an all-solid-state beam steering realized by integrated optical phased array (OPA) is one of the key components in the LiDAR system. In this review, we first introduce the basic principle of OPA for beam steering. Then, we briefly review the detailed advances of different solutions such as micro-electromechanical system OPA, liquid crystal OPA, and metasurface OPA, where our main focus was on the recent progress of OPA in photonic integrated chips. Finally, we summarize the different solutions and discuss the challenges and perspectives of all-solid-state beam steering for LiDAR.

## 1. Introduction

The principle of light detection and ranging (LiDAR) is to emit an optical electromagnetic signal and detect the echo-signal of the targets. Compared to traditional microwave radar, LiDAR, working at optical wavelengths, could provide a higher angular resolution with the advantage of a vastly improved diffraction limit [1]. As the source of LiDAR, lasers have high coherence and high directivity. Thus, LiDAR has the potential to achieve long-haul detection, which has extensive application prospects in fields such as autonomous drive, terrain/ocean mapping, free space optical communication, etc. [2,3,4,5,6,7].

Generally, LiDAR can be divided into two categories: mechanical LiDAR and solid-state LiDAR. Most commercially available LiDAR systems are mechanical LiDAR [8], which obtains beams by steering through mechanical control components such as mirrors. The mechanical LiDAR can realize a large field-of-view (FOV) by controlling rotary assembly [9]. However, such mechanisms limit the scanning speed and decrease reliability [10]. Suffering from the complex and precise assembly and calibration processes, it is hard to satisfy the requirements of non-inertia and miniaturization in many applications [11]. Therefore, all solid-state beam steering systems have attracted the attention of many researchers. The beam steering via optical phased array (OPA) technology, which originated from the mature microwave phased array theory, has been a research hotspot of late. Various beam steering techniques based on OPA have been exploited, mainly including the micro-electromechanical system (MEMS) OPA, liquid crystal (LC) OPA, metasurface (meta) OPA, and photonic integrated chip (PIC) OPA.

In this paper, the state-of-the-art of all-solid-state OPA in recent years is reviewed, mainly focusing on the performances of the beam steering range, spatial resolution, and scanning rate. In Section 2, we introduce the principle of beam steering realized by OPA. In Section 3, we mainly review the progress in the fields of MEMS OPA, LC OPA, meta OPA, and PIC OPA based on different semiconductor platforms. In Section 4, we compare the pros and cons of different types of OPA and discuss the development prospects of solid-state beam steering.

## 2. Principle

The far electric field distribution of OPA can be calculated from the near electric field distribution, according to Fresnel diffraction theory [12,13]:

(1) $$e(x_{1},y_{1})=\frac{je^{-jkz_{0}}}{\lambda z_{0}}e^{-\frac{jk}{2z_{0}}(x_{1}{}^{2}+y_{1}{}^{2})}\iint e(x_{0},y_{0})e^{-\frac{jk}{2z_{0}}(x_{0}{}^{2}+y_{0}{}^{2})}e^{j\frac{2\pi }{\lambda z_{0}}(x_{0}x_{1}+y_{0}y_{1})}dx_{0}dy_{0}$$

where *e*(*x*_0_, *y*_0_) is the near electric scalar field in *x*_0_*y*_0_-plane; *e*(*x*_1_, *y*_1_) is the far electric scalar field in *x*_1_*y*_1_-plane; *z*_0_ is the distance between *x*_0_*y*_0_-plane and *x*_1_*y*_1_-plane; and *k* is the free-space wave vector.

According to microwave theory, the far field radiation pattern of an antenna located at (*x_0_*, *y_0_*) in the *x*_0_*y*_0_-plane is given by:

(2) $$e(x_{1},y_{1})=F(\theta_{x},\theta_{y})\frac{e^{-jkR}}{R}$$

where *θ_x_* and *θ_y_* is the lateral beam steering angle and the longitudinal beam steering angle, respectively, which can be calculated using the standard spherical angles *θ* and *φ* (*θ* is the angle with the z-axis and *φ* is the angle between the projection on the *xy*-plane and the *x*-axis) [13] according to thee formulas of sin*θ_x_* = sin*θ* cos*φ* and sin*θ_y_* = sin*θ* sin*φ*. *F*(*θ_x_*, *θ_y_*) are the radiation patterns and *R*^2^ = *(x_1_ − x_0_)*^2^ *+ (y_1_ − y_0_)*^2^ + *z*_0_^2^.

**Figure 1:**
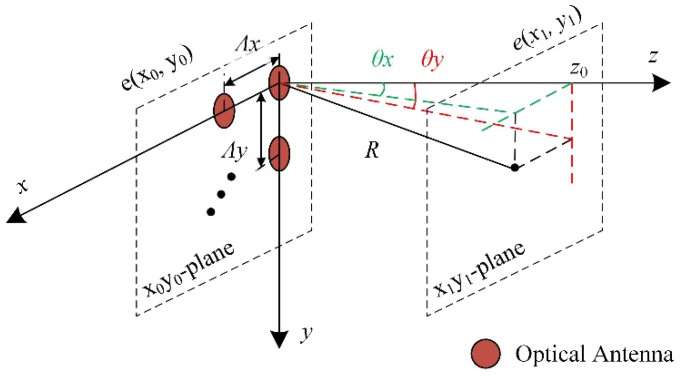
Schematic of the OPA.

For a *M* × *N* OPA with a lateral and longitudinal pitch of *Λ_x_* and *Λ_y_* (as shown in Figure 1), respectively, the far field is:

(3) $$e(x_{1},y_{1})=\sum_{m=0}^{M-1}\sum_{n=0}^{N-1}A_{mn}\cdot F_{mn}(\theta_{x},\theta_{y})\frac{e^{-jkR_{mn}}}{R_{mn}}\cdot e^{-jk(m\cdot \Lambda_{x}\sin\theta_{x}-\beta_{m}{}_{x})}\cdot e^{-jk(n\cdot \Lambda_{y}\sin\theta_{y}-\beta_{ny})}$$

where *F_mn_*(*θ_x_*, *θ_y_*) is the radiation patterns of antenna located at different position; *R_mn_*^2^ = (*x*_1_ *− mΛ_x_*)^2^ + (*y*_1_ *−nΛ_y_*)^2^ + *z*_0_^2^; *A_mn_* is field amplitude; and *β_mx_*, *β_ny_* are the phase differences.

For the far-field region, *F_mn_*(*θ_x_*,*θ_y_*) can be approximated well to *F*_00_(*θ_x_*,*θ_y_*) and *R_mn_* can be *R*_00_. For the uniform OPA, the field amplitude *A_mn_* can be considered as 1. Therefore, the far-field radiation pattern of OPAs is a far-field radiation pattern of individual antenna multiplied by the array factor. Achieving beam steering by OPA, there should be a phase difference between adjacent antennas. Here, *β_mx_* = *m*Δ*φ_x_*/*k*, *β_ny_* = *n*Δ*φ_y_*/*k*. The normalized array factor *T*(*θ_x_*,*θ_y_*) can be obtained by extracting the common factors and normalizing Equation (3):

(4) $$T(\theta_{x},\theta_{y})=\frac{\sin\left[M\left(k\Lambda_{x}\sin\theta_{x}-\Delta \varphi_{x}\right)/2\right]}{M\sin\left[\left(k\Lambda_{x}\sin\theta_{x}-\Delta \varphi_{x}\right)/2\right]}\cdot \frac{\sin\left[N\left(k\Lambda_{y}\sin\theta_{y}-\Delta \varphi_{y}\right)/2\right]}{N\sin\left[\left(k\Lambda_{y}\sin\theta_{y}-\Delta \varphi_{y}\right)/2\right]}$$

**Figure 2:**
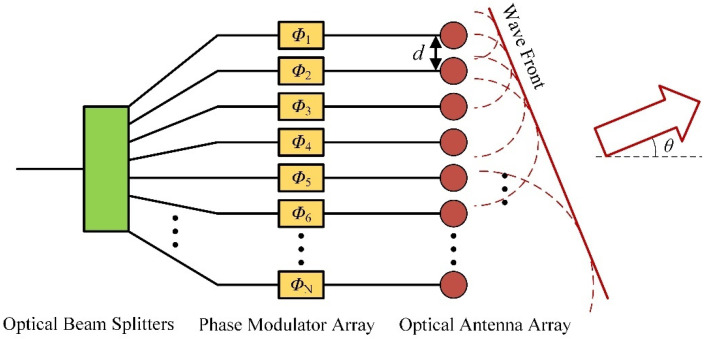
The schematic diagram of the one-dimension optical phased array.

The normalized array factor has the same expression form in two directions, so one only needs to study the law of one dimension and the other dimension is the same. Generally [14,15,16], an OPA chip consists of optical beam splitters, a phase modulator array, and an optical antenna array, as shown in Figure 2. The power of the laser source is distributed to the unit of the phase modulator array and the optical antenna array by the beam splitters. The optical wavefront is controlled by the phase modulator array, thus the far-field beam is steered. Quantitatively, according to Equation (4), the normalized array factor will be maximum when the following equation is satisfied:

(5) $$\frac{k\Lambda \sin\theta -\Delta \varphi }{2}=q\pi$$

where *q* = (0, ±1, ±2, …). When the absolute value of *q* increases, the phase difference between adjacent antennas becomes larger, which will make the phase modulator consume more power. Consider that the central principal maximum, the relationship between the phase difference of the adjacent phase unit Δ*Φ*, and the beam steering angle *θ* are shown in the following equation [17]:

(6) $$\theta =\arcsin\left(\frac{\Delta \Phi \cdot \lambda }{2\pi \cdot d}\right)$$

where *λ* is the wavelength and *d* is the interval of antennas, as shown in Figure 2.

**Figure 3:**
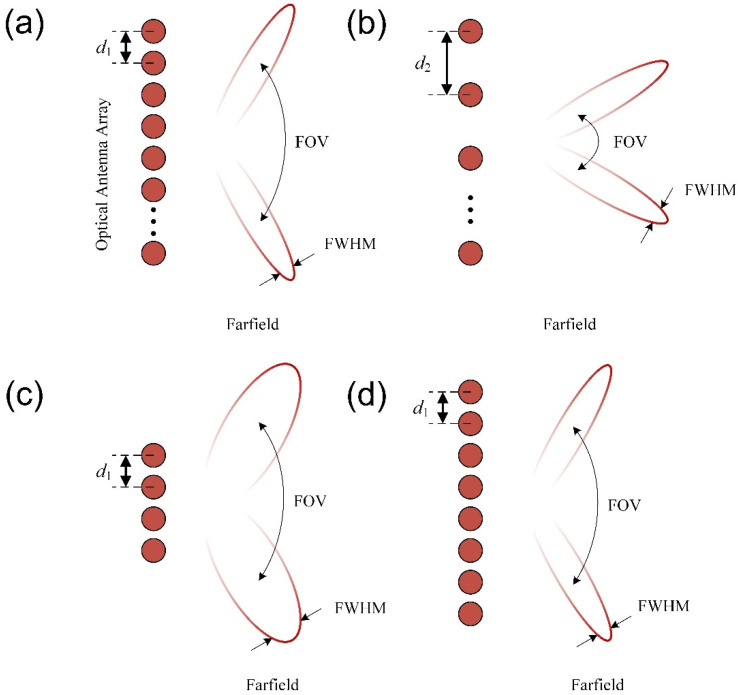
The schematic diagram of the far-field beam with (**a**) densely spaced antennas and (**b**) widely spaced antennas with the same antenna aperture; (**c**) small optical apertures, and (**d**) large optical apertures with the same antenna interval.

As shown in Figure 3a, when Δ*Φ* = π, the central principal maximum is located at arcsin(*λ*/2*d*) and the -1th order lobe is at -arcsin(*λ*/2*d*). The FOV is given by:

(7) $$\Delta \theta_{FOV}=2\cdot \arcsin\left(\frac{\lambda }{2d}\right)$$

When *Nd >> λ,* the full width at half maximum (FWHM) is given by:

(8) $$\Delta \theta_{FWHM}\approx \frac{0.886\lambda }{Nd\cdot \cos\theta }$$

Here, we will show the influence of different intervals and aperture sizes on the FOV and FWHM, which can be calculated according to Equations (7) and (8). By comparing Figure 3a,b, one can see that the densely spaced antenna array could obtain a larger FOV than the widely spaced one. The FOV could reach 180° when the antenna interval *d* was smaller than *λ*/2, independent of the phase difference Δ*Φ*. Figure 3c,d shows the antenna arrays with the same interval, but with different aperture sizes, where one can see that a larger aperture size could achieve a narrower beam width.

## 3. OPAs for Beam Steering

### 3.1. MEMS-OPA

Mechanical LiDAR is one of the most common types of LiDAR, and has the characteristics of remote detection and large FOV [18,19]. However, such types of LiDAR are bulky, power-hungry, and vulnerable to mechanical shock [20]. Although many miniaturization efforts have been reported, it is still difficult to meet the requirements of robotic mobile platforms [11].

**Figure 4:**
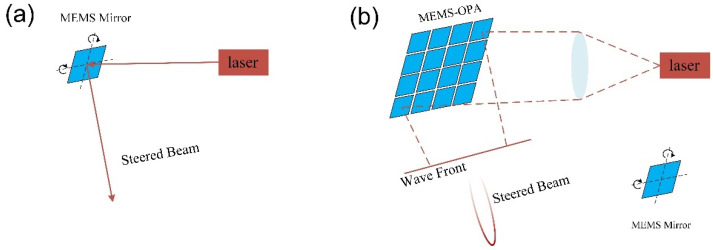
The schematic diagram of the (**a**) resonant MEMS mirror and (**b**) MEMS-OPA.

The appearance of the MEMS has had a great impact on micromanufacturing and microsystems, and provides an alternative scheme for LiDAR to reduce costs, reduce the energy consumption, and increase the scanning speed [8]. MEMS mirrors can modulate, light, and control phase, which have already found enormous commercial success in projectors, displays, and fiber optic communications [21,22,23]. Two-dimensional (2D) resonant MEMS mirrors, actuated by the electrothermal [24], electrostatic [25], electromagnetic [26], or piezoelectric effect [27], have been used to achieve beam steering [28], as shown in Figure 4a. There is a trade-off between the optical aperture and scanning speed for resonant MEMS mirrors. A large optical aperture is needed to obtain high-resolution scanning, which will lead to a large mass and limit the rotation speed.

A MEMS-OPA could solve this challenge by dividing a large aperture into small micromirrors with significantly reduced inertia [29]. A MEMS-OPA can realize beam steering by combining phased array technology with a MEMS system, as shown in Figure 4b. The phase difference Δ*Φ* can be provided by tilting the individual micromirror array.

In 2014, Megens et al. proposed a MEMS antenna of high-contrast grating (HCG). The antenna realized >99% reflectivity using a subwavelength grating patterned on the polysilicon [30]. The beam steering angle of the 32 × 32 MEMS-OPA was ±2° and the beam divergence was 0.14°. The response time was as low as 3.8 μs. The larger scale MEMS-OPA, having 160 × 160 independent phase shifters within an aperture of 3.1 mm × 3.2 mm, was fabricated by Wang et al. The beam steering angle can be extended to 6.4° × 4.4°, and the beam divergence can be compressed to 0.042° × 0.031° [31]. The MEMS-OPA has a greatly reduced mass compared to the traditional MEMS rotating mirror and can achieve a modulation bandwidth over 500 kHz at low driving voltage. The FOV of the MEMS-OPA can be extended with large-scale integration, but the fabrication difficulty is also increased. The FOV can also be extended by a lens-assisted system, where the total FOV is 9.14° × 9.14°. Meanwhile, the beam divergence can also be reduced by about 4.4 times compared to the one without a lens [32]. However, the large interval *d* of the MEMS array fundamentally limits the beam steering range/FOV.

### 3.2. LC-OPA

With the development of the liquid crystal materials and control technology, LC-OPA emerged rapidly after the first liquid crystal material-based OPA was demonstrated by McManamon et al. [33]. The feasibility of the liquid crystal application in the all-solid-state LiDAR was preliminarily verified.

**Figure 5:**
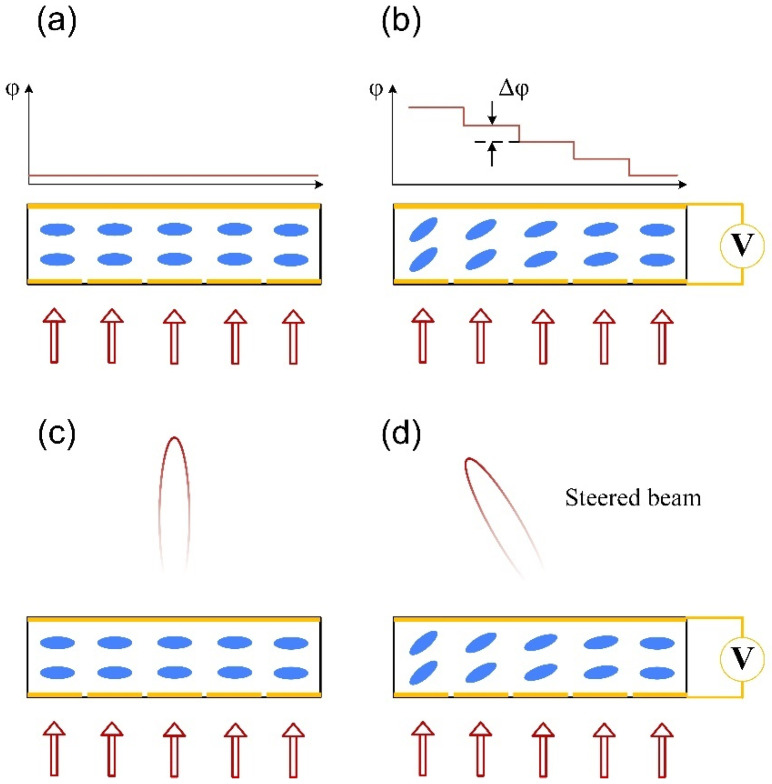
The schematic diagram of the LC-OPA. The phase retardation distribution (**a**) before and (**b**) after applying voltage. The far-field beam (**c**) before and (**d**) after applying voltage.

The basic principle of LC-OPA is shown in Figure 5. LC-OPA has the advantage of high birefringence [34]. As shown in Figure 5a, the phase relation between array units remains constant without applying voltage. With the applied voltage, as shown in Figure 5b, one can introduce local changes in the refractive index by changing the orientation of the liquid crystal [35]. Therefore, the phase difference between the array units can be adjusted and beam steering be achieved accordingly.

The LC-OPA has many advantages such as low drive voltage, compact size, high precision, and non-inertia. However, the angular resolution is difficult to achieve due to the limitation of the pixel pitch, and the fringe-field effect arising in the neighboring pixels [34]. Furthermore, the scanning speed is limited due to the time needed for the molecular reorientation [36]. Hence, researchers have mainly focused on how to increase the response speed and reduce the beam divergence and steering precision. In 2009, Engström et al. introduced a ferroelectric liquid crystal material-based spatial light modulator to increase the response speed. The rise/fall times were less than 200 μs, and the beam steering range was ±9° [37]. In 2013, Wang et al. proposed a liquid crystal vernier OPA integrating a LC wedge and a LC grating electronically and realized a fine steering precision without affecting the steering range [38]. The experimental results showed that the precision was better than 2 μrad. In 2019, to realize high angular resolution and low beam divergence in the LC-OPA, Qin et al. [34] introduced two nonparallel blazed gratings with a special included angle, achieving multiple diffractions for the incident light, to compress the steered angle of the incident light. The angular resolution of beam steering (~10 μrad) was improved six times compared to that without the use of the double-grating configuration experimentally. However, problems remain in achieving a wide angle, high efficiency, and continuous beam steering by LC devices [35]. For example, the beam steering angle is also limited by the large interval of the LC array. The LC-OPA has great potential to be applied to modern holography, head-worn displays, and so on [35,36,39], but the scanning angle and speed should be further improved to meet the requirements in driverless cars and other artificial intelligence fields.

### 3.3. Meta-OPA

**Figure 6:**
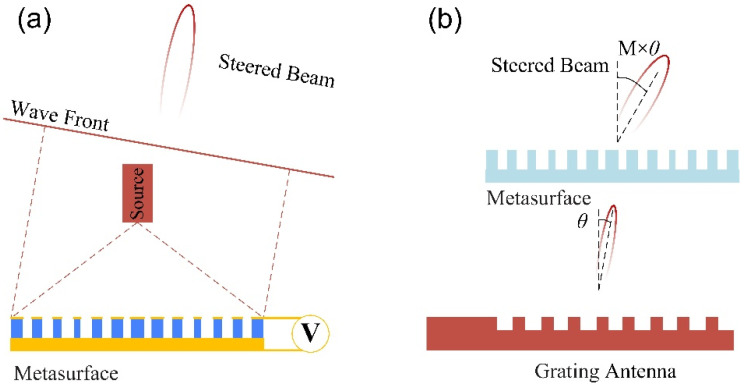
The schematic diagram of the meta-OPA: (**a**) normal meta-OPA; (**b**) meta-assisted PIC-OPA.

Metasurfaces, featuring ultrathin and flat form factor as well as high design flexibility [40,41], can manipulate the amplitude, phase, and polarization state of light with extreme freedom [42]. There are two ways to achieve beam steering with metasurfaces. The first one is by applying an external electric field to control the material properties of the metasurfaces and manipulate the wavefront [42,43,44,45,46] (named as normal meta-OPA), as shown in Figure 6a. In 2021, Ai et al. [42] designed a metasurface structure consisting of graphene ribbons, a dielectric spacer, and metal substrate. The researchers obtained single-, double-, and triple-beam steering by designing the graphene ribbons and controlling the applied voltage on them. With only one phase gradient, a deflection angle of 41.98° was obtained with TM polarized incident light at a frequency of 12.32 THz.

Another method is to enlarge the FOV of PIC-OPA assisted by metasurfaces (named meta-assisted PIC-OPA). As shown in Figure 6b, the metasurface performs like an ultra-thin lens, which can enlarge the beam steering angle *θ* by *M* times [47,48,49]. Lee et al. proposed and demonstrated a bidirectional grating antenna PIC-OPA integrated with a miniaturized all-dielectric metasurface doublet formed on a glass substrate atop the antenna array. The steering efficiency was confirmed to be boosted by a factor of ~3.1. The steering angle was up to 30° with the wavelength from 1530 to 1595 nm [49]. However, for normal meta-OPA, the phase modulation is often generally accompanied by intensity modulation [42,44], which affects the side mode suppression ratio. In terms of the meta-assisted PIC-OPA, the beam divergence increased simultaneously with the improvement in the FOV.

### 3.4. PIC-OPA

PIC has the great potential to integrate the key components of OPA such as the laser, beam splitters, phase modulator arrays, antennas, and photodetectors on a single chip [50,51,52]. The phase modulators can utilize the thermo-optic [53,54] or electro-optic effect [55] to tilt the far-field wavefront, which can obtain the steered beam.

In 1972, Dr. Meyer realized a one-dimensional OPA using lithium tantalate (LiTaO_3_) crystal for the first time, which verified the concept of OPA and provided a new technical approach to obtain beam steering [56]. The PIC-OPA has been demonstrated in different material platforms such as III–V, silicon nitride (SiN), and silicon.

The III–V material is one of the significant platforms. A one-dimensional PIC-OPA in the InP platform has been proposed and demonstrated [57,58]. In 2021, Komatsu et al. proposed a 100 channel OPA and p-i-n phase modulators were fabricated to control the optical phase [57]. The operating wavelength of the InGaAs/InP OPA can also be extended to the mid-infrared wavelength band, of which the atmospheric window band (3–5 um) can meet the applications in long-range LiDAR [17]. Most importantly, the III–V platform has the capability of realizing monolithic integration [59]. Passive and active devices such as a high-power laser source, amplifier, and high speed modulator can be integrated monolithically.

With careful design and fabrication, the SiN waveguide loss can be ultra-low [60]. SiN also suffers from less nonlinear losses with respect to Si [61], which allows waveguide to operate at high laser power. Since the optical transparency window can be down to a wavelength of 500 nm, silicon nitride has become an attractive platform for the PIC-OPA in the visible band [62,63]. Poulton et al. proposed a large-aperture visible PIC-OPA at 635 nm with a spot size of 0.064° × 0.074°, of which the far-field beam is static [64]. A 2D PIC-OPA in the near-infrared band was proposed by Tyler et al., where the π phase-shift power consumption was measured to be 87.6 mW [63]. The power consumption was relatively large due to the low thermo-optical coefficient, the magnitude of which was 10^−5^/K.

With the rapid development of CMOS technology in the past decades, silicon-based optoelectronic integration has the advantages of monolithically large-scale integration, low power consumption, and low cost. It is considered as one of the most promising technologies [65,66]. The various structures and functions of silicon photonic devices have been proposed theoretically and experimentally [67,68]. Therefore, an OPA-based on silicon photonic integration technology provides a new solution for LiDAR. Many schemes have been proposed in the aspects of enlarging the beam steering range, improving the spatial resolution, and reducing the power consumption.

**Figure 7:**
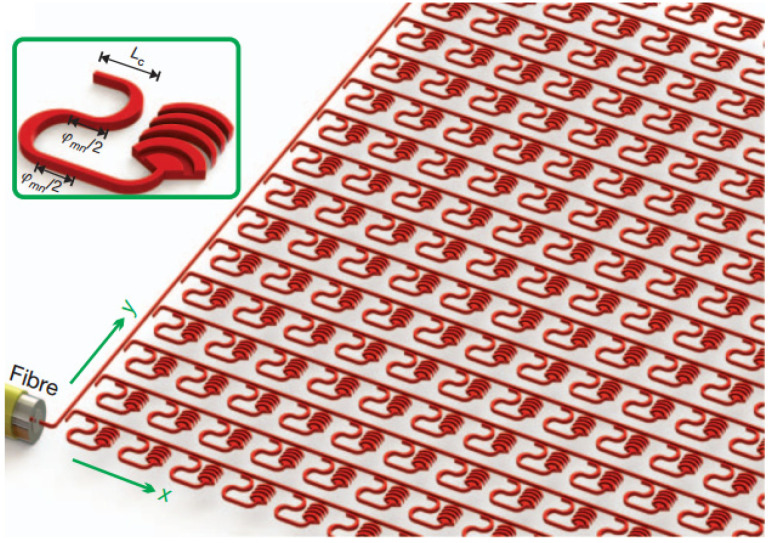
The architecture of the large-scale integrated 2D optical phased antenna array from [69]. Reproduced with permission from [69].

In order to obtain 2D beam steering with a silicon-based PIC-OPA, one way is to use a 2D antenna array arrangement. Researchers from MIT studied OPA with 2D nanoantenna structures on silicon, as shown in Figure 7. Nano-antennas of 4096 were integrated in one chip and successfully applied to image the MIT logo in 2013 [69]. To compress the side lobe, they utilized the intensity apodization in the antenna, and a 24° beam steering range with a beam divergence of 1.6° was achieved [70]. The beam steering angle was also limited by the large interval of the antenna array. The sparse arrangement OPA can compress the grating lobes and achieve the grating-lobe-free field-of-view (FOV)-to-beam width ratio of 16°/0.8° [71]. For the large scale antenna of the OPA in planar, the scalability suffers from waveguide routing and power consumption. Ashtiani et al. proposed a method that used cross waveguides to route the input light to each grating antenna, and reduced the number of phase shifters from N^2^ to 2N [72].

Due to the limitation of the beam steering range and complexity of the two-dimensional antenna array OPA, researchers have proposed another 2D OPA scheme by combining the wavelength tuning and phase tuning. The beam steering angle *φ*, which varies with wavelength, is determined by the following equation:

(9) $$\sin\varphi =\frac{\Lambda n_{eff}-\lambda }{n_{b}\Lambda }$$

where *Λ* is the grating period; *n_eff_* is the effective index of the guiding mode; *n_b_* is the background index; *λ* is the wavelength.

**Figure 8:**
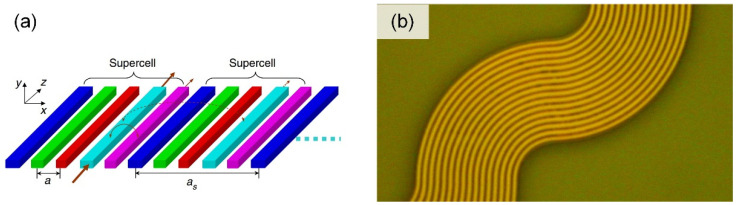
(**a**) The schematic of a waveguide superlattice array from [73]; (**b**) a microscopy image of the curved waveguide array [76]. Reproduced with permission from [73].

In the steering dimension of phase modulation, the sparse aperiodic antenna is used to suppress the grating lobes, but at the cost of the main lobe power [71]. Theoretically, the narrower the antenna spacing, the larger the field of view without grating lobes can be obtained. When the antennas are spaced at half of the operating wavelength, there is only one main lobe at ±90° FOV. However, when the spacing decreases, the crosstalk between the waveguides/antennas increases dramatically. The key to achieving large beam steering range in the phase tuning dimension is to reduce the crosstalk. One possible solution is to introduce the effective refractive index mismatch between the adjacent waveguides of the waveguide array. In 2015, Song et al. proposed a waveguide superlattice and conducted a detailed theoretical analysis in a silicon platform [73], as shown in Figure 8a. The waveguides with different widths were interleaved so that there would be an effective refractive index mismatch between the neighboring waveguides. The waveguides with the same widths were separated far from each other and the coupling strength was low. Hence, such waveguide superlattices could enable high-density waveguide integration at a half-wavelength pitch with low crosstalk. By using the waveguide superlattice, the antenna array with a half-wavelength pitch was demonstrated in PIC-OPA [74,75]. The beam steering range of ±80° was measured experimentally [74]. In addition, a curved waveguide can also be used to introduce an effective refractive index mismatch and achieve a dense waveguide array [76,77], as shown in Figure 8b. A large beam steering range achieved by introducing an effective refractive index mismatch has been verified in 1D-OPA with only phase tuning, but it is difficult to integrate the wavelength tuning. Due to the phase mismatch, it is necessary to solve the problems of the difference in beam steering angle and the difference in wavelength tuning efficiency caused by the effective refractive index mismatch of different waveguides.

In the other steering dimension, beam steering can be achieved by tuning the operating wavelength according to Equation (9). However, the beam steering range is usually ∼14°, which is limited by the wavelength tuning range of the light source (~100 nm) and the bandwidth of the power splitters [78,79,80]. To solve this problem, researchers have proposed multi-line OPA. In 2020, Zhang et al. integrated four/eight OPAs with different output grating emitter arrays in a single chip to increase the wavelength tuning efficiency [81]. The eight-line OPA was 19.04° with wavelengths ranging from 1520 nm to 1540 nm.

**Figure 9:**
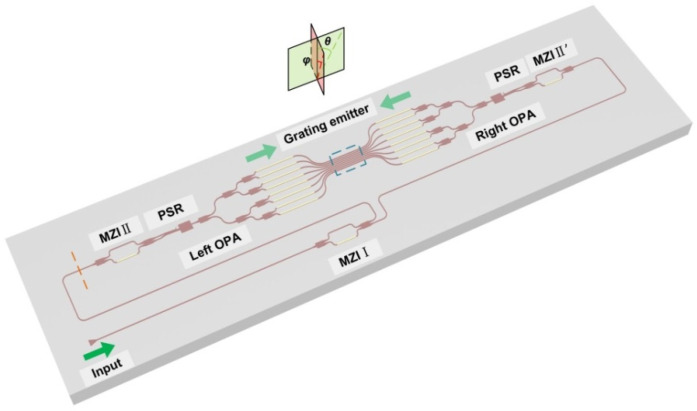
A schematic of the bi-directional dual polarization multiplexed OPA [85].

The combination of wavelength division multiplexing and polarization/mode multiplexing technology can effectively improve the optical communication capacity [82,83]. Thus, multi-dimensional multiplexing technology is also promising to increase the steering angle. Generally, due to the significant birefringence, the silicon-on-insulator (SOI) based waveguide is usually polarization dependent. According to Equation (2), a beam with different polarization states, introducing different effective refractive index, will be steered to different angles. The polarization multiplexing OPA is proposed in detail, and the key components are optimized carefully [84]. The total steering angle was up to 28.2° with the wavelength scanning range from 1500 nm to 1600 nm. Compared to the traditional single-polarization OPA, the wavelength tuning efficiency was improved twice to 0.28°/nm. Our group also proposed a dual polarization and bi-directional OPA, as shown in Figure 9, to further increase the FOV in the wavelength dimension. The wavelength tuning efficiency was further doubled to 0.55 °/nm, with only one waveguide grating antenna array [85].

Aside from the beam steering angle, beam divergence is also another important performance of the OPA. Higher spatial resolution could be achieved by compressing the beam divergence, which requires a larger aperture.

The aperture in the phase tuning dimension can be improved by increasing the number of antennas or the pitch of antennas. Poulton et al. demonstrated an OPA with a phase element of 8192 and antenna apertures of 8 mm × 5 mm. Combined with wavelength tuning, 2D beam steering with a beam divergence of 0.01° × 0.039° was obtained [86]. Hutchison et al. designed and fabricated a 128-channel OPA with an appropriate sparse non-uniform antenna spacing to suppress the grating lobes and demonstrated a small beam divergence of 0.142° in the phase tuning dimension [87].

**Figure 10:**
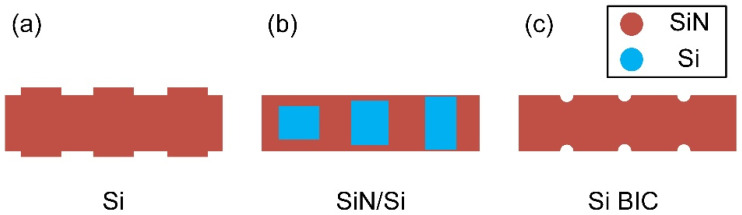
The schematic diagram of the (**a**) sidewall grating antenna; (**b**) SiN/Si grating antenna; (**c**) BIC-based silicon waveguide grating antenna.

The beam divergence of the wavelength tuning dimension mainly depends on the effective aperture. The light intensity in the antenna decays exponentially when it propagates along the waveguide grating. Weaker grating strength guarantees a larger effective aperture and higher resolution [88,89]. To improve the effective aperture in the wavelength tuning dimension, grating perturbation should be weakened. One possible solution is to reduce the corrugation of the grating structures. As shown in Figure 10a, Miller et al. fabricated the sidewall gratings with an ultra-small feature size of 10 nm, which achieved a 1 mm propagation length. The beam divergence is as narrow as 0.15° in the state-of-the-art [90]. Utilizing SiN grating perturbation instead of an air one, as shown in Figure 10b, can also shrink the refractive index difference. The uniform emission intensity across the long emission length (>1 mm) can be obtained by varying the grating width and duty cycle [91]. In 2020, our group proposed the extremely weak diffraction intensity by harnessing the bound state in the continuum (BIC) in the all-silicon dielectric platform, as shown in Figure 10c. Based on the experimental results, it could be indicated that an ultralong propagation length >1 cm and a narrow far-field divergence ~0.027° can be achieved [92].

## 4. Discussion

Compared with the conventional beam steering by mechanical assembly, MEMS have a great impact on micromanufacturing and microsystems, and provides an alternative scheme for LiDAR to reduce the energy consumption/cost and increase the scanning speed. However, there is a trade-off between the optical aperture and scanning speed for the resonant MEMS mirror. The MEMS-OPA, which realizes beam steering by combining phased array technology with the MEMS mirror, can solve the trade-off problem and reduce the inertia. Changing the orientation of the liquid crystal or manipulating the wavefront by the metasurface is another way to realize beam steering. However, the large interval of the MEMS/LC array fundamentally limits the beam steering range/FOV and modulation rate. Furthermore, the MEMS-OPA, LC-OPA, and meta-OPA, which find it difficult to integrate the laser source, are commonly used in spatial light modulators. Therefore, it is urgent to develop all-solid-state and fully integrated chips for beam steering. The PIC-OPA has emerged with the development and trend.

The OPA performance with the representative technical solutions is shown in Table 1. Theoretically, the beam steering range and beam divergence/FWHM of OPA affect the FOV and spatial resolution of the LiDAR system, respectively. The improvement in the beam steering range enables the LiDAR to have a larger FOV, and the reduction in the beam divergence/FWHM enables the target details to be better distinguished. In terms of the beam steering range, the PIC-OPA has gradually expanded its advantages over MEMS-OPA, LC-OPA, and meta-OPA with a special arrangement of antenna arrays such as waveguide superlattice, grating-lobe compression technology, or multi-dimensional multiplexing technology. Resolution can also be improved with the increasing aperture size of antennas, which will put forward new demands on large/very large-scale integration. Generally, there are three key problems. First, the footprint of large-scale on-chip LiDAR is mainly limited by the overall size of the electrode arrangement and the voltage control unit. The greater the number of phased array units, the more difficult the electrode arrangement and the voltage control. Multilayer electrode distribution is promising in solving this problem. Second, the power consumption is also a key problem that needs to be solved within large-scale integration, which boosts the research into high-efficiency phase shifters. Third, due to the on-chip waveguide loss and the nonlinear effect of material, the PIC-OPA is difficult to apply in long-haul scanning and detection. Fortunately, novel platforms such as SiN/Si or LiNbO_3_/Si multi-layer materials are potential alternatives.

In summary, MEMS-OPA, LC-OPA, meta-OPA, and PIC-OPA have significant advantages such as their compact size, high precision, non-inertia, and so on. CMOS-compatible PIC-OPA is one of the most promising solutions for all-solid-state and miniaturized LiDAR. The significant components of the OPA such as the laser, beam splitters, phase modulator arrays, antennas, and photodetectors can be integrated on a single chip. Since there are many problems in the commercial application of PIC-OPA, the realization of all-solid-state and miniaturized LiDAR with high performance parameters such as a large beam steering angle and high spatial resolution deserve further research. The progress of silicon monolithic integration and advanced laser/detector technology will promote the development of OPA LiDAR. It can be predicted that mature LiDAR will have commercial application in the field of artificial intelligence and communication.

## Figures and Tables

**Table 1:** The performance comparison of the recently demonstrated OPA.

Type			Ref (Year)	The Number of Antenna	Scanning Range (°)	Resolution (°)
MEMS-OPA			[93] (2014)	32 × 32	±2	0.14
			[31] (2019)	160 × 160	6.6 × 4.4	0.042 × 0.031
LC-OPA			[94] (2017)	/	40	/
			[95] (2016)	/	/	2
Meta-OPA			[42] (2021)	/	41.98	/
			[44] (2021)	/	7.68	2
			[49] (2021)	32	30	1.8 × 0.7
PIC-OPA		2D antenna-OPA ^1^	[70] (2014)	8 × 8	24 × 24	1.6 × 1.6
			[71] (2019)	128	16 × 16	0.8 × 0.8
		1d antenna-OPA ^2^	[57] (2021)	1 × 100	8.88 × /	0.11 × /
			[87] (2016)	1 × 128	80 ×17	0.14 × 0.14
			[74] (2018)	1 × 64	160 × /	2.9 × /
			[1] (2019)	1 × 512	56 × 15	0.04 × /
			[96] (2021)	1 × 128	140 × 19.23	0.021 × 0.1
			[85] (2022)	1 × 16	77.8 × 54.5	3.6 × 2

^1^ 2D antenna-OPA—only with phase tuning; ^2^ 1D antenna-OPA—with phase tuning plus X tuning (X represents wavelength or polarization).
